# Perspectives on Triple-Negative Breast Cancer: Current Treatment Strategies, Unmet Needs, and Potential Targets for Future Therapies

**DOI:** 10.3390/cancers12092392

**Published:** 2020-08-24

**Authors:** Gagan K. Gupta, Amber L. Collier, Dasom Lee, Richard A. Hoefer, Vasilena Zheleva, Lauren L. Siewertsz van Reesema, Angela M. Tang-Tan, Mary L. Guye, David Z. Chang, Janet S. Winston, Billur Samli, Rick J. Jansen, Emanuel F. Petricoin, Matthew P. Goetz, Harry D. Bear, Amy H. Tang

**Affiliations:** 1 Leroy T. Canoles Jr. Cancer Research Center, Department of Microbiology and Molecular Cell Biology, Eastern Virginia Medical School, Norfolk, VA 23501, USA; GuptaGK@EVMS.EDU; 2 DeWitt Daughtry Family Department of Surgery, Surgical Oncology, University of Miami/Jackson Memorial Hospital, University of Miami Miller School of Medicine, Miami, FL 33131, USA; amber.collier@jhsmiami.org; 3 Department of Medicine, Internal Medicine, H. Lee Moffitt Cancer Center and Research Institute, University of South Florida, Tampa, FL 33620, USA; dlee7@usf.edu; 4 Dorothy G. Hoefer Foundation, Sentara CarePlex Hospital, Newport News, VA 23666, USA; rahoefer@sentara.com; 5 Sentara Cancer Network, Sentara Healthcare, Norfolk, VA 23507, USA; mlguye@sentara.com; 6 Surgical Oncology, Cancer Treatment Centers of America—Comprehensive Care and Research Center Phoenix, 14200 W Celebrate Life Way, Goodyear, AZ 85338, USA; Vasilena.Zheleva@ctca-hope.com; 7 Department of OB/GYN, University of Kentucky College of Medicine, University of Kentucky, Lexington, KY 40536, USA; lauren.vanreesema@uky.edu; 8 Department of Molecular and Cell Biology, UC Berkeley, Berkeley, CA 94720, USA; AngelaTangTan@Berkeley.edu; 9 Sentara Surgery Specialists, Sentara CarePlex Hospital, Newport News, VA 23666, USA; 10 Virginia Oncology Associates, 1051 Loftis Boulevard, Suite 100, Newport News, VA 23606, USA; David.Chang@USOncology.com; 11 Breast Pathology Services, Pathology Sciences Medical Group, Department of Pathology, Sentara Norfolk General Hospital (SNGH), Norfolk, VA 23507, USA; JSWINSTO@sentara.com (J.S.W.); bxsamli@sentara.com (B.S.); 12 Department of Public Health, North Dakota State University, Fargo, ND 58102, USA; rick.jansen@ndsu.edu; 13 Center for Applied Proteomics and Molecular Medicine, School of Systems Biology, George Mason University, Manassas, VA 20110, USA; epetrico@gmu.edu; 14 Departments of Oncology and Pharmacology, Mayo Clinic Breast Cancer Specialized Program of Research Excellence (SPORE), Women’s Cancer Program, Mayo Clinic Cancer Center, Mayo Clinic, Rochester, MN 55905, USA; goetz.matthew@mayo.edu; 15 Departments of Surgery and Microbiology & Immunology, Division of Surgical Oncology, Massey Cancer Center, Virginia Commonwealth University, Richmond, VA 23298, USA; harry.bear@vcuhealth.org

**Keywords:** triple-negative breast cancer (TNBC), EGFR/K-RAS/SIAH signaling pathway, pathologic incomplete responders (pIR), tumor-driving signaling pathways in TNBC, neoadjuvant chemotherapy (NACT), residual cancer burden (RCB), concurrent ACT regimen (Adriamycin, Cytoxan, and Taxotere), sequential ACT regimen (AC-T), chemo-resistance, tumor recurrence, clinical diagnostics, prognostics, improved patient survival

## Abstract

Triple-negative breast cancer (TNBC), characterized by the absence or low expression of estrogen receptor (ER), progesterone receptor (PR), and human epidermal growth factor receptor (HER2), is the most aggressive subtype of breast cancer. TNBC accounts for about 15% of breast cancer cases in the U.S., and is known for high relapse rates and poor overall survival (OS). Chemo-resistant TNBC is a genetically diverse, highly heterogeneous, and rapidly evolving disease that challenges our ability to individualize treatment for incomplete responders and relapsed patients. Currently, the frontline standard chemotherapy, composed of anthracyclines, alkylating agents, and taxanes, is commonly used to treat high-risk and locally advanced TNBC. Several FDA-approved drugs that target programmed cell death protein-1 (Keytruda) and programmed death ligand-1 (Tecentriq), poly ADP-ribose polymerase (PARP), and/or antibody drug conjugates (Trodelvy) have shown promise in improving clinical outcomes for a subset of TNBC. These inhibitors that target key genetic mutations and specific molecular signaling pathways that drive malignant tumor growth have been used as single agents and/or in combination with standard chemotherapy regimens. Here, we review the current TNBC treatment options, unmet clinical needs, and actionable drug targets, including epidermal growth factor (EGFR), vascular endothelial growth factor (VEGF), androgen receptor (AR), estrogen receptor beta (ERβ), phosphoinositide-3 kinase (PI3K), mammalian target of rapamycin (mTOR), and protein kinase B (PKB or AKT) activation in TNBC. Supported by strong evidence in developmental, evolutionary, and cancer biology, we propose that the K-RAS/SIAH pathway activation is a major tumor driver, and SIAH is a new drug target, a therapy-responsive prognostic biomarker, and a major tumor vulnerability in TNBC. Since persistent K-RAS/SIAH/EGFR pathway activation endows TNBC tumor cells with chemo-resistance, aggressive dissemination, and early relapse, we hope to design an anti-SIAH-centered anti-K-RAS/EGFR targeted therapy as a novel therapeutic strategy to control and eradicate incurable TNBC in the future.

## 1. Introduction

Breast cancer is the most commonly diagnosed cancer in women world-wide, and metastatic breast cancer (MBC) is the second leading cause of cancer-related deaths in American women [1,2,3]. There are more than 3.5 million women who have been diagnosed with breast cancer in the United States alone [4]. In 2020, an estimated 276,480 new cases of female breast cancer will be diagnosed, and 42,170 MBC patients are expected to succumb to their disease [1]. Breast cancer is classified into four distinct molecular subtypes based on the expression profile of ER, PR, and/or HER2 receptors [5,6,7]. Increased early screening, high-resolution imaging technology, and the design of effective chemotherapy, radiation, targeted, and immunotherapy sequences have extended patients’ lives significantly [8,9,10,11,12]. Currently, more than 98% of patients with early-stage breast cancer survive for 10–15 years or longer [13,14,15]. Although the death rate from female breast cancer dropped by 40% from 1989 to 2017, the five-year survival rates for localized, regional, and distant diseases are at 99%, 86%, and 27% (https://www.cancer.org/cancer/breast-cancer/understanding-a-breast-cancer-diagnosis/breast-cancer-survival-rates.html). Overall, the five-year survival rate for all stages combined together in breast cancer remains at 90% in 2020. Thus, there remains a significant number (10%) of breast cancer patients who continue to succumb to chemo-refractory metastatic and inoperable disease. This subgroup of patients represents an unmet need that could be addressed with precision medicine, multi-omics data profiling, multi-dimensional drug integration, and curative therapeutic innovations in the clinic [16,17,18,19].

The prognosis for patients with locally advanced and metastatic disease remains poor [1,20]. MBC displays dynamic, unpredictable, and rapidly evolving genetic diversity and biological heterogeneity of the disease burden and tumor microenvironment (TME) [9,11]. MBC remains an incurable disease despite many diagnostic and therapeutic advances in the past 30 years [16]. Developing effective diagnostics and beneficial therapies to treat MBC and reduce MBC mortality remains an urgent priority. Concentrated efforts to develop new targeted therapies based on major tumor vulnerabilities within key tumor-driving signaling pathways, multi-omics systems biology, and synergistic implementation of immune checkpoint blockade therapies against MBC tumors have offered new avenues of improving outcomes for chemo-resistant, relapsed, and metastatic breast cancer.

## 2. Triple Negative Breast Cancer (TNBC)

TNBC represents 15% of all breast cancers in the United States, and is characterized by the absence of ER, PR, and HER2 receptor expression [10,12,14,21,22]. TNBC is the most aggressive phenotypic subtype of breast cancer [22,23,24,25,26,27]. Pathologic features of TNBC include higher mean tumor size, tumor grade, and proliferation index at diagnosis compared with non-TNBC tumors [28]. TNBC is nearly twice as common in African American women than in Caucasian women, and more common in premenopausal women and *BRCA1/2* mutation carriers [18,23,24,29,30,31,32,33,34,35,36,37]. TNBC has the worst outcomes of all breast cancer subtypes with a five-year overall survival (OS) of 78.5%, even when adjusting for age, disease stage, race, tumor grade, and receipt of adjuvant chemotherapy [5,6,22,37,38]. Depending on their response to initial chemotherapy, one in three TNBC patients will develop tumor recurrence, which typically occurs within the first three years of initial diagnosis, and persistently, one in five TNBC patients will succumb to their metastatic disease in less than five years [21,22,26]. The five-year survival rates for localized, regional, and metastatic TNBC are 91%, 65%, and 11%, respectively (https://www.cancer.org/cancer/breast-cancer/understanding-a-breast-cancer-diagnosis/types-of-breast-cancer/triple-negative.html). The dismal prognosis of high-risk, locally advanced, and metastatic TNBC highlights an unmet need for an improved survival in this subtype.

Another reason for the poor outcomes associated with TNBC is the lack of effective targeted therapies which are commonly used to treat ER^+^/PR^+^ and HER2^+^ breast cancer subtypes [21,22,23,39]. Due to the low or absent expression of ER, PR, and HER2 receptors, endocrine therapies such as selective estrogen receptor modulators (SERMs) and aromatase inhibitors, or anti-HER2 targeted monoclonal antibody treatments like trastuzumab are ineffective in treating TNBC [5,40,41]. As a result, standard cytotoxic chemotherapy remains the backbone of systemic therapy in TNBC [7,10,12,38,42]. TNBC tumors have shown a higher pathologic complete response (pCR) rate (approximately 30–40%) to chemotherapies (doxorubicin, docetaxel, 5-fluorouracil, platinum drugs, and/or cyclophosphamide), compared to non-TNBC tumors [21,23,43,44]. The pCR of TNBC post-neoadjuvant chemotherapy (NACT) predicts long-term survival [45,46,47,48,49]. Patients whose tumors exhibit a pathologic incomplete response (pIR) with residual disease post-NACT, are more likely to suffer early recurrence and reduced survival [50,51,52,53]. Notably, by measuring residual disease after NACT, the risk of developing a future life-threatening distant event can be accurately quantified [54,55] and TNBC patients with high-risk residual disease are now commonly considered for additional adjuvant chemotherapies, including capecitabine, post-operatively [7,56,57].

Further attempts to classify TNBC into distinct subtypes based on unique tumor/TME cellular signatures and mRNA expression profiles may provide relevant information about the molecular drivers, actionable therapeutic targets, and effective therapy selection [58,59,60,61,62,63,64]. While there is controversy about the number of TNBC subtypes, it is well accepted that there are at least two–three major subtypes, including the basal and luminal androgen receptor (LAR) subtypes and likely the mesenchymal subtype [61,62,65,66]. The proposed immunomodulatory subtype may simply represent an effect of the tissue microenvironment, and not a specific TNBC subtype after adjusting for tumor infiltrating lymphocyte (TIL) levels. Additional sub-classifications of the basal-like (BL1 and BL2), and mesenchymal (M) subtypes are more controversial [62,65,67]. Notably the LAR subtype is enriched with hormone signaling, steroid synthesis, androgen/estrogen metabolism, and overexpression of androgen receptors (AR) [61,62,66,68].

Based on the PAM50 gene expression profile, 78.6% of TNBC have significant overlap with the basal-like molecular subtype [5,66,69]. The remaining gene expression profiles of TNBC (21.4%) may be further sub-classified as normal-like (7%), HER2-enriched (7.8%), luminal B (4.4%), and luminal A (2.2%) [66]. The claudin-low subset of TNBC is a particularly aggressive subtype [70,71]. Even though the assessment and characterization of TNBC into molecular subtypes is not currently performed clinically on a routine basis, these sub-classifications based on unique cellular signatures and global RNA expression profiles may provide therapeutic insights for each specific subset of TNBC patients. By targeting TNBC subtype-specific tumor drivers, additional targeted therapies may augment the standard of care (SOC) for each unique subtype of TNBC patients [39,44]. Using the molecular-based TNBC sub-classifications, new subtype-specific tumor vulnerabilities and actionable drug targets may be identified to apply and re-purpose FDA-approved drugs to treat chemo-resistant, late-stage, and metastatic TNBC tumors [72,73,74].

## 3. Current TNBC Treatment Paradigms

At the time of diagnosis, clinicopathological parameters such as patient age, TNM (tumor size, lymph node status, metastasis), stage, tumor grade, histology, and molecular subtype of breast tumors are commonly used to support medical decision-making in selecting and prescribing the effective treatment regimens. In a move towards enhanced precision medicine, the eighth edition of the American Joint Committee on Cancer (AJCC) added prognostic biomarkers to the traditional anatomic staging classification in TNBC [75,76,77]. In designing the optimal treatment sequencing and therapy combinations, there are many considerations with respect to balancing the risks and benefits in treating early-stage and low-risk TNBC. Over-treatment may lead to chemo-toxicity without benefit and under-treatment may lead to early relapse and poor outcomes. In contrast, high-risk and locally advanced TNBC necessitate aggressive treatment with a variety of chemotherapy schedules and drug combinations. Since cytotoxic chemotherapy is often the only available systemic option to treat TNBC in order to reduce and prevent tumor relapse and systemic metastasis, a vast majority of TNBC patients with high-risk and locally advanced disease have no choices but to endure standard chemotherapies as prescribed [21,22,78,79,80]. Ineffective chemotherapy and chemo-toxicity increases the burden of treatment, and often leads to undesirable side effects and long-term adverse health consequences, adversely impacting the patient’s quality of life.

Early-stage TNBCs with tumor sizes ≤ 0.5 cm (T1a) or between 0.6–1.0 cm (T1b) without lymph node involvement (N0) generally have a good prognosis [81]. A retrospective study demonstrated five-year relapse-free survival (RFS) of 75–88.6% and five-year distant recurrence-free survival (DRFS) greater than 95.2% in 143 patients with T1a,bN0 TNBC treated without adjuvant chemotherapy [82]. Another study involving 363 patients with T1a,bN0 TNBC compared the five-year distant recurrence-free survival (DRFS) between patients who were treated with adjuvant chemotherapy to those who were not. Untreated T1a and T1b TNBC had a five-year distant recurrence-free survival (DRFS) of 93% (n = 74) and 90% (n = 94), as compared to treated T1a and T1b TNBC which had a distant recurrence-free survival (DRFS) of 100% (n = 25) and 96% (n = 170), respectively. These authors concluded that there was no significant benefit to the addition of chemotherapy for TNBC tumors that were less than 1 cm [81]. Additionally, a recent analysis of centrally confirmed and systemically untreated TNBC with T1, N0 disease (n = 182), found the five-year invasive disease-free survival (IDFS) was as follows: [T1a: 82.5% (95% confidence interval (CI), 62.8–100; T1b: 67.5% (95% CI, 51.9–87.8); and T1c: 67.3% (95% CI, 54.9–82.6)] [83]. Due to the lack of prospective randomized data for managing T1, N0 TNBC, the decision to use or withhold chemotherapy remains a clinical judgment for the individual oncologist and patient preference.

Systemic chemotherapy is the backbone therapy recommended for TNBC patients with a tumor size > 1 cm with or without lymph node (LN) metastases. Standard chemotherapy regimens for TNBC are usually based on a combination of anthracyclines, alkylators, and taxanes [84]. Anthracycline and taxane therapies have also been found to improve outcomes of TNBC patients [85,86]. The ABC trial determined that the addition of anthracycline to docetaxel and cyclophosphamide therapy resulted in a small, though significant improvement in the four-year invasion disease-free survival (IDFS) for TNBC patients, raising it from 88.2% to 90.7% with a *p* value of 0.04 [85]. Additionally, the adjuvant breast cancer trial GEICAM 9906 tested the benefits of adding weekly paclitaxel after the completion of adjuvant fluorouracil, epirubicin, and cyclophosphamide (FEC) in LN-positive breast cancer. The addition of eight weekly paclitaxel doses to standard FEC chemotherapy (FEC-P) decreased the likelihood of tumor relapse by 47% and improved the seven-year DFS by 18% compared to FEC alone [86].

Chemotherapy can be given to treat high-risk and early-stage TNBC in the neoadjuvant and/or adjuvant setting [22,87,88]. Given no differences in survival between the adjuvant and neoadjuvant settings, neoadjuvant chemotherapy (NACT) is now considered the standard approach to treat high-risk TNBC to reduce tumor burden and evaluate chemo-efficacy prior to surgical resection [71,88,89,90,91,92,93,94,95]. NACT provides a number of distinct advantages, including: (1) potential to reduce the scope of surgery for the primary breast tumors (e.g., segmental instead of total mastectomy); (2) reduced scope of axillary node resection (e.g., sentinel node biopsy versus complete axillary node dissection); (3) time to consult plastic surgeons and genetic counselors prior to surgery; and (4) most significantly, an opportunity to assess tumor response, prognosis, and the potential need for additional or adjuvant treatments.

While high-resolution imaging is commonly used to track tumor response and follow patients receiving NACT [96,97,98,99,100,101], radiologic assessments are imperfect predictors of pathologic response at surgery. pCR is the most important prognostic clinical parameter in TNBC [101,102]. The complete disappearance of invasive cancer post-NACT has been strongly linked to favorable outcomes [46,88]. For example, patients who have achieved pCR at the primary tumor site and axillary lymph nodes (defined as absence of invasive cancer in the breast and regional lymph nodes or ypT0/Tis, N0) post-NACT have the longest disease-free survival and significantly improved OS [46,88,103,104,105,106,107]. In contrast, pIR forecasts an increased risk of early tumor relapse and a significantly shorter disease-free survival (DFS) with chemo-resistant and progressive disease post-NACT [46,90,108,109]. The pIR in partial responders with an increased amount of residual diseases, such as moderate and extensive residual cancer burden (RCB II-III), is prognostic and predictive of poor outcome and reduced survival [54,55,110,111,112]. To further reduce tumor recurrence and metastatic spread, a significant portion of TNBC pIR patients with the RCB II-III classification will elect to undergo additional rounds of adjuvant chemotherapies as their health and performance status permit. The CREATE-X trial showed that addition of adjuvant capecitabine improved the rate of disease-free survival (DFS) by 13.7% and OS by 8.5% after preoperative chemotherapy in TNBC [56]. The additional adjuvant chemotherapy is now considered standard therapy by NCCN to treat TNBC pIR patients with residual diseases post-NACT.

Although pCR is associated with the best outcomes, this is not an “all or none” relationship because some TNBC pCR patients still develop tumor relapse years later [101,102,113,114]. The RCB was developed by the MD Anderson Cancer Center using a formula based on tumor size, invasive cancer cellularity, and nodal status post-NACT [54,55]. Tumors assessed by the RCB are numerically classified as RCB 0-III, with the higher the RCB score or classification indicating a higher likelihood of subsequent recurrence, metastatic spread, and increased mortality from breast cancer. As a result, the high-risk RCB classification provides a continuous projection of the risk for recurrence for pIR patients post-NACT [54,55,110,111,112]. Furthermore, TNBC outcomes have also been correlated with quantitative assessment of immune response, such as enumerating TIL within the residual tumors post-NACT [83,115,116,117,118,119,120,121,122]. The addition of carboplatin to anthracycline plus taxane-based regimens has been tested in several trials in the neoadjuvant setting, demonstrating increased pCR rates but also greater hematologic toxicity [123,124,125]. Only the Geparsixto trial, which used a non-standard chemotherapy approach, showed early improvement in DFS [123,126]. The other two trials, CALGB 40603 and BrighTNess, did not demonstrate improved outcomes despite the increased pCR rates [124,125]. Extended analysis of the German trial found significantly better DFS [Hazard Ratio (HR) 0.56; *p* = 0.022] with the addition of carboplatin and a modest (6%) though not statistically significant improvement in OS [125]. Interestingly, high TIL infiltration in the pre-treatment tumors was associated with the greatest benefit from the addition of carboplatin to NACT [122,126,127].

## 4. Prognosis and Treatment Heterogeneity in TNBC

Despite the strong correlations between pathologic responses, pCR and pIR, and RCB with clinical outcomes, the outcomes of TNBC patients with pIR tumors may vary widely. One unmet need is distinguishing which pIR patients will remain disease-free and which of them will relapse following SOC chemotherapy. Many TNBC patients with similar clinical and pathological presentations often respond very differently to standard chemotherapies [31,88,128]. Therefore, accurately predicting and anticipating which partial responders will relapse and which ones will stay in remission post-NACT remains an unresolved problem in clinical oncology. Advanced imaging technology and RCB classifications are unable to predict tumor recurrence and metastatic potential with certainty for individual pIR patients. Although the identification and classification of high-risk RCB tumors post-NACT is important, it remains insufficient, since we still cannot differentiate between chemo-resistant residual tumor clones, particularly those at distant sites, that are still growing, from chemo-sensitive residual tumor remnants that have stopped growing post-NACT. As a result, developing new, precise, and high-resolution prognostic molecular biomarker(s) is needed to stratify and differentiate high-risk from low-risk residual TNBC tumors post-NACT. Recently, it was found that the detection of circulating tumor DNA and circulating tumor cells in liquid biopsy post-NACT is associated with tumor recurrence in TNBC [129]. New prognostic and predictive biomarkers are needed to provide real-time quantitative and interactive tumor information, thereby assisting oncologists to select and guide second-line treatments in hopes of eradicating chemo-resistant TNBC [129,130]. Such biomarkers may also have the potential to serve as new drug targets for subsequent alternate therapies [131]. Chemo-radiation, and targeted therapies are known to select for resistant tumor clones if complete eradication is not achieved with first-line and second-line therapies [39,71,92]. It is of paramount importance that a majority of pIR patients with residual diseases should receive precision-driven, tailored, and curative adjuvant therapy in a timely fashion at frontline settings to control and eradicate chemo-resistant metastases, independent of RCB classification post-NACT [22,40].

The survival benefit of treating high-risk TNBC patients with concurrent or sequential chemotherapies is comparable whether patients are treated with either neoadjuvant or adjuvant chemotherapy [87]. However, there are multiple advantages in using NACT. NACT is interactive, quantitative, evidence-driven, and a preferred option compared to adjuvant chemotherapies, which are largely blind without the primary tumor as a surrogate marker post-surgery. Adjuvant-treated TNBC patients can have heightened anxiety and chronic stress due to the uncertainty of not knowing whether the prescribed post-operative chemotherapy has been effective in achieving a complete eradication of all the invisible disseminated tumor cells. TNBC recurrence both loco-regional or distant metastases generally may not be curable despite all the available second-line and/or third-line therapeutic regimens and advanced treatment arsenals. In contrast, NACT offers distinct clinicopathological benefits by directly measuring the tumor response of each individual TNBC tumor in a paired fashion pre- and post-NACT. Finally, adjuvant-treated TNBC patients miss an opportunity to receive additional evidence-based treatments known to prolong survival (e.g., capecitabine) based on initial response to standard chemotherapy. Such a quantitative, interactive, comparative, and precision-driven platform would be invaluable for risk-stratifying TNBC patients, quantifying chemo-efficacy, forecasting early relapse, and predicting patient survival. There is a distinct advantage in the early identification and close interrogation of the disseminated and residual chemo-resistant tumor cells responsible for early tumor relapse and systemic metastases post-NACT. As such, NACT offers a valuable window of opportunity for a data-driven molecular monitoring and quantification platform of real-time TNBC tumor responses as a prelude to accurate molecular prediction of tumor relapse, outcome, and survival in the clinic [13,95,131,132]. Based on dynamic tumor responses and major tumor vulnerabilities revealed in real time, it opens the possibility to develop new actionable targets and novel therapies that can be added in tandem to eradicate chemo-resistant and invasive residual tumor cells post-NACT.

Lastly, the survival rates for chemo-resistant, relapsed, and metastatic TNBC patients have not improved significantly over the past 30 years [92]. High-risk and locally advanced TNBC tumors have high inter- and intra-tumor heterogeneity, which becomes more pronounced in chemo-resistant, relapsed, and metastatic settings. Chemo-resistant TNBC has consistently challenged our ability to design better targeted therapies to save more patients with progressive and metastatic disease [133,134,135,136,137,138,139]. Ultimately, there is a pressing need to identify the major TNBC vulnerability, target the conserved and key TNBC-driving signaling pathways, and develop new innovative strategies to identify and control multidrug-resistant, relapsed, and late-stage TNBC, preferably before metastatic deposits become clinically detectable and/or often incurable in the clinic [19,140,141,142].

## 5. Newly FDA-Approved Targeted Therapies for TNBC

### 5.1. Immune Checkpoint Blockade Therapies

Immune checkpoint inhibitors targeting programmed death receptor-1 (PD-1) and programmed death ligand-1 (PD-L1) have shown some promise in treating advanced and metastatic TNBC in combination with standard chemotherapy [79,143,144,145,146,147,148,149]. PD-L1 is predominantly expressed on infiltrating immune cells while only 5% of TNBC express PD-L1. PD-1 is often expressed on TILs, especially T cells. When PD-L1 binds to PD-1, it produces an inhibitory signal that results in T-cell suppression [79,150,151,152]. PD-L1 expression in TNBC occurs predominantly on infiltrating immune cells, and some tumor cells [143,153,154,155,156]. The presence of increased or densely clustered TIL or expression of PD-L1/PD-1 immune checkpoint molecules is usually associated with a better prognosis, increased tumor immunity, and identifies potential candidates for immune checkpoint blockade therapy [151,153,157,158,159]. PD-L1 is expressed in approximately 40% of TNBC tumors and TNBC-associated tumor stromal and infiltrating immune cells in the TME, which is more frequent than for non-TNBC tumors [79,83,160]. For example, ER^+^-luminal mammary tumors are rarely associated with high levels of TILs or PD-L1 expression [161]. As a result, PD-L1 has become a promising new therapeutic target because of its high prevalence and increased expression in metastatic TNBC (mTNBC) [145,146].

The phase 3 IMpassion130 trial (NCT02425891) tested the benefits of adding atezolizumab, an anti-PD-L1 monoclonal antibody, to nab-paclitaxel chemotherapy as compared to nab-paclitaxel alone as a first-line therapy for 902 mTNBC patients who were partitioned in a 1:1 ratio of 451 patients in each treatment arm [143,144,154]. In an unselected mTNBC cohort, the addition of atezolizumab to nab-paclitaxel improved progression-free survival (PFS) modestly (7.2 months vs. 5.5 months, respectively), but did not significantly improve OS (21.3 months vs. 17.6 months) in the atezolizumab/nab-paclitaxel arm when compared to the chemotherapy (nab-paclitaxel)-alone arm. However, in a pre-specified analysis of a PD-L1-positive TNBC cohort (PD-L1 positivity is defined by PD-L1 expression on tumor-infiltrating immune cells that cover ≥ 1% of the tumor area), the addition of atezolizumab to nab-paclitaxel significantly improved median PFS of 7.4 months versus 4.8 months, respectively (HR 0.60; 95% CI, 0.48–0.77; *p* < 0.0001), and a larger benefit on OS of 25 months versus 15.5 months, respectively (HR, 0.62; 95% CI, 0.45–0.86) when compared to the chemotherapy (nab-paclitaxel)-alone arm. Therefore, approximately 40% of TNBC patients with PD-L1 expression on infiltrating immune cells in the tumors are likely to benefit from the addition of an anti-PD-L1 antibody like atezolizumab. Of note, there were increased treatment-related adverse effects due to the addition of atezolizumab, as 15.9% of patients discontinued either the atezolizumab or nab-paclitaxel compared to 8.2% of patients receiving nab-paclitaxel and the placebo [143,144]. Based on the IMpassion130 trial results, the FDA granted accelerated approval of atezolizumab (Tecentriq) to treat PD-L1-positive unresectable locally advanced and metastatic TNBC in combination with nab-paclitaxel (Abraxane) on March 8, 2019 [143,144].

In the neoadjuvant setting, the phase III KEYNOTE-522 trial (NCT03036488) studied the addition of pembrolizumab (Keytruda), an anti-PD-1 monoclonal antibody, to neoadjuvant chemotherapy and continued adjuvant chemotherapy in 1174 untreated stage II or III TNBC patients who were partitioned in a 2:1 ratio of 784 patients in the pembrolizumab–chemotherapy group and 390 patients in the placebo–chemotherapy group. The pCR rate was 64.8% (95% CI, 59.9–69.5) in the pembrolizumab–chemotherapy group versus 51.2% (95% CI, 44.1–58.3) in the placebo–chemotherapy group (estimated treatment difference, 13.6%; 95% CI, 5.4–21.8; *p* < 0.001) [162,163]. After a median follow-up of 15.5 months, the disease progression rate was recorded as 7.4% in the pembrolizumab–chemotherapy group, and 11.8% in the placebo–chemotherapy group (HR 0.63; 95% CI, 0.43–0.93) [162,163]. In contrast to the IMpassion130 trial, the addition of pembrolizumab to standard chemotherapy in the KEYNOTE-522 trial demonstrated improvements in pCR, independent of PD-L1 expression status [162]. Additional prospective studies on pembrolizumab have yielded promising results. Preliminary data from the KEYNOTE-355 (NCT02819518) study on patients with untreated locally recurrent inoperable or metastatic TNBC that expressed PD-L1 with a combined positive score (CPS) ≥ 10 tumors showed that the addition of pembrolizumab to chemotherapy significantly improved PFS compared to chemotherapy alone (9.7 vs. 5.6 months, respectively). OS and the significance of the addition of pembrolizumab in TNBC patients with low CPS >1 tumor are still being investigated (KEYNOTE-355 Abstract—Cortes et al., (2020) Randomized, double-blind, phase III study of pembrolizumab + chemotherapy versus placebo + chemotherapy for previously untreated locally recurrent inoperable or metastatic triple-negative breast cancer. https://ascopubs.org/doi/abs/10.1200/JCO.2020.38.15_suppl.1000).

Despite these exciting developments, promising efficacy, and rapid FDA approval of incorporating immuno-chemotherapy to treat unresectable, locally advanced, relapsed, and metastatic TNBC, the success of atezolizumab/pembrolizumab is still modest measured by an improved five-year survival. Chemo-, radiation, and targeted therapy may be used to prime, synergize, and invigorate PD-1 inhibition in TNBC [146]. The successes of multidrug combination and correct treatment sequencing are often incremental and anecdotal in eliciting a robust antitumor immune response to kill off the immunologically “cold” mTNBC consistently and reliably. Treatment strategies to convert immunologically “cold” tumors into immunologically “hot” ones remain a clinical challenge and an unmet need in TNBC, since we aim to recapitulate and reproduce the remarkable successes reported in 15–45% of late-stage melanoma and non-small cell lung cancer patients whose previously incurable tumors were able to achieve durable response to immune checkpoint blockade therapy in combination with chemo-, radiation, and targeted therapies to induce and maximize immune cell-mediated tumor cell killing in vivo and significantly extend the long-term survival [164,165,166,167,168,169,170,171,172,173,174].

### 5.2. PARP Inhibitors

BRCA1/2 are well-known tumor suppressor genes whose loss of function mutations are associated with early-onset, increased familial inheritance, sporadic incidence, tumor aggression, and poor outcomes in breast cancer [175,176,177,178]. Recently, poly-ADP-ribose polymerase (PARP) inhibitors identified via synthetic lethal screens were clinically tested, and received FDA approval—all in record time [179,180,181,182,183]. Currently, BRCA1/2-mutant mammary tumors are being treated with anti-PARP targeted therapies, including approximately 19.5% of TNBC [184]. Normal *BRCA*1/2 proteins are responsible for homologous double-stranded dsDNA repair with the help of additional protein partners, including PARP enzymes. The inhibition of PARP1 or PARP2—the most abundant PARP enzymes—leads to the accumulation of irreparable breaks of both single-stranded and double-stranded DNA and cytotoxic PARP-DNA complexes [180,185,186,187]. As a result, TNBC tumors carrying *BRCA* mutations and/or other similar DNA repair pathway mutations are sensitive to PARP inhibitor therapy [179,188,189]. The OlympiAD trial (NCT02000622) is a phase 3 randomized study to examine the efficacy of olaparib, a PARP1 inhibitor, for patients with metastatic, germline *BRCA* mutated, HER2-negative breast cancer, and who had received no more than two previous lines of chemotherapy or treatments of physician’s choice [190]. The trial results showed that olaparib monotherapy provided a significant benefit over standard chemotherapy, i.e., median progression-free survival (PFS) was 2.8 months longer and the risk of disease progression or death was 42% lower in patients who received olaparib, compared to those who received standard therapy of capecitabine, eribulin, or vinorelbine [190,191,192]. Olaparib was generally well-tolerated with minimal side effects and acceptable toxicity. However, an important finding was that there was no statistically significant improvement in OS with olaparib compared to standard chemotherapy in this cohort. In this cohort of HER2-negative metastatic breast cancer patients with a germline *BRCA* mutation, a subset of TNBC and ER^+^/PR^+^ MBC patients were studied and an improved PFS was reported in this OlympiAD trial [190,191,192]. The EMBRACA trial studied the efficacy of talazoparib, another PARP inhibitor, on advanced breast cancer patients with germline *BRCA1/2* mutations and who had been previously treated with chemotherapy. This study similarly showed that patients who took talazoparib had improved PFS compared to patients who received single-agent chemotherapy. The positive responses were consistently documented in a subset of TNBC patients’ germline *BRCA1/2* mutations. Of note, PARP inhibitors are typically well-tolerated drugs and can be added to standard chemotherapy to synergistically treat mammary tumors with germline mutations with either high, intermediate, or low penetrance in the homologous recombination pathway, and in the single-strand and double-strand DNA break repair machinery in hopes of improving the clinical outcome and quality of life of TNBC patients with germline mutations in *BRCA1, BRCA2*, *PALB2, RAD51, p53,* and *CHEK2* [188,193,194,195,196,197,198,199,200,201].

### 5.3. Anti-Trop2 Antibody Drug Conjugate Therapy in TNBC

Trophoblast cell-surface antigen (Trop-2) is a glycoprotein overexpressed in many epithelial cancers as a pro-growth signal [202]. Sacituzumab govitecan-hziy is an anti-Trop-2 antibody conjugated to an active metabolite of irinotecan (SN-38) [203,204]. This antibody drug conjugate inhibits topoisomerase activity and its DNA binding, prevents ligation of cleaved DNA strands, results in double-strand DNA breaks, triggers cell death, and blocks DNA replication in tumor cells [202,203]. The effects of sacituzumab govitecan-hziy have been studied on heavily pretreated mTNBC patients [204,205,206]. Sacituzumab govitecan-hziy is well tolerated and induced an improved response rate and median PFS (33.3% and 5.5 months, respectively) compared to standard chemotherapy treatment (10–15% and 2–3 months, respectively) [206]. The phase 3 ASCENT trial (NCT02574455) was a confirmatory randomized study designed to validate the safety and efficacy data of sacituzumab govitecan previously reported in a Phase 2 study of heavily pretreated patients with metastatic TNBC [206]. Recently, the phase 3 ASCENT study of metastatic TNBC was halted due to compelling and convincing evidence of impressive drug efficacy after this antibody drug conjugate significantly improved progression-free survival (PFS), overall survival (OS), objective response rate (ORR), and durable objective responses in heavily pretreated mTNBC patients without brain metastasis. In this advanced mTNBC cohort, Sacituzumab govitecan demonstrated a statistically significant improvement in PFS compared to standard chemotherapy (HR, 0.41; 95% CI, 0.32–0.52). The mTNBC patients that received sacituzumab govitecan-hziy had a PFS of 5.6 months (95% CI, 4.3–6.3), compared to that of 1.7 months (95% CI, 1.5–2.6) for patients who received chemotherapies of physician’s choice (*p* < 0.0001) [206]. In April 2020, Sacituzumab govitecan-hziy (Trodelvy) received accelerated FDA approval for heavily pretreated and advanced mTNBC based on these promising and exciting results [205,206]. Since then, Trodelvy^®^ has become the very first antibody drug conjugate to be approved for patients with relapsed or refractory mTNBC who have failed two prior chemotherapies (https://www.immunomedics.com/our-company/news-and-events/immunomedics-announces-positive-results-from-phase-3-ascent-study-of-trodelvytm/).

Due to the clinical success of PD-L1/PD-1 inhibitors, PARP inhibitors, and anti-Trop-2 antibody drug conjugates, these targeted drugs have received FDA approval and now warrant clinical consideration in the treatment of selected subsets of TNBC patients with the aforementioned clinical indications. Importantly, Trodelvy has demonstrated a clear clinical benefit in a heavily pretreated and advanced mTNBC population. Furthermore, mTNBC patients are being tested for the expression of PD-L1 in TILs and/or germline *BRCA1/2* mutations to determine if they qualify for one of the new targeted therapies. PD-1 inhibitors (pembrolizumab) and PD-L1 inhibitors like atezolizumab in combination with chemotherapies are being considered for administration at the frontline settings to treat locally advanced, recurrent, and metastatic TNBC as early as possible, given the promising results of the IMpassion130 and KEYNOTE-355 trials [143,144]. Despite these amazing promises and tangible successes, approximately half of mTNBC patients’ tumors that express PD-L1 in infiltrating immune cells and even a smaller minority of TNBC patients carry germline *BRCA1/2* mutations. Therefore, many TNBC patients would not benefit from these recently FDA-approved targeted therapies.

## 6. Emerging Targeted Therapies in TNBC

There are several emerging therapies and repurposed drugs targeting tumor-driving signaling pathways in TNBC, including epidermal growth factor (EGFR/HER1) antibodies, PI3K/AKT/mTOR, and angiogenesis inhibitors, androgen receptor (AR) antagonists, and estrogen receptor beta (ERβ) agonists [39,207,208,209]. These drugs are currently still under clinical investigation with limited or mixed results, and therefore they are not a part of standard of care (SOC) therapy.

### 6.1. EGFR Targeted Therapy in TNBC

EGFR activation/amplification is detected in approximately 25–50% of TNBC [210,211,212,213]. In theory, EGFR inhibition by anti-EGFR monoclonal antibodies like cetuximab and/or EGFR small molecule inhibitors should be effective in the treatment of EGFR-driven TNBC. Unfortunately, multi-centered clinical trials have not shown cetuximab to be an effective therapy for TNBC. For instance, the TBCRC 001 trial tested the effects of cetuximab alone and cetuximab plus carboplatin therapy on stage IV TNBC patients whose heavily pretreated tumors progressed and metastasized despite multiple rounds of chemotherapy. The study found that cetuximab alone and cetuximab plus carboplatin produced responses in only 6% and 16% of patients, respectively [39,214]. In a subset of the TNBC patient population that underwent serial biopsy, only a minority of patients demonstrated minimal EGFR pathway inhibition after receiving cetuximab alone or cetuximab plus carboplatin. This result suggested that cetuximab was largely ineffective in inhibiting EGFR pathway activation in TNBC, likely as a result of compensatory signaling pathway activation downstream of the EGFR receptor. Instead of being diminished, the EGFR activation signal was sustained by signaling bifurcation, cancer network crosstalk, and compensatory pathway activation, as there are several intertwined major cellular signaling pathways that are tightly regulated by active EGFR signals [214]. Given these negative results, cetuximab is not currently recommended for the treatment of TNBC with EGFR overexpression.

### 6.2. VEGF Targeted Therapy in TNBC

Vascular endothelial growth factor (VEGF) is the most important angiogenic factor in breast cancer since it stimulates tumor cell proliferation and growth as well as new vessel formation in growing tumors. VEGF expression is often higher in TNBC compared to non-TNBC, and increased VEGF expression is associated with poor outcomes independent of tumor size, nodal status, and histological grade [215]. Clinical studies of bevacizumab, an anti-VEGF antibody, have shown improvements in PFS but insignificant improvements in OS in TNBC. The BEATRICE trial evaluated the outcomes of TNBC patients treated with adjuvant bevacizumab and chemotherapy as compared to chemotherapy alone [216,217]. No significant improvement in the three-year IDFS and/or OS for patients treated with bevacizumab compared to chemotherapy alone was found (83.7% vs. 82.7%, respectively) [217,218]. Another study tested the benefits of adding bevacizumab to chemotherapy as first-line treatment of HER2-negative metastatic breast cancer in a large cohort of 2447 patients [219]. The authors reported that patients treated with bevacizumab and chemotherapy had improved median PFS compared to chemotherapy alone (8.1 months vs. 5.4 months, respectively) and marginally improved median OS (18.9 months vs. 17.5 months, respectively) [219]. In the neoadjuvant setting, one study found that bevacizumab added to chemotherapy increased the pCR rate for TNBC, but another study found that the increase in pCR achieved with the addition of bevacizumab was confined to non-TNBC tumors [220,221,222]. However, neither study showed any significant improvement in five-year survival for TNBC, consistent with several other neoadjuvant trials [103,124,223,224]. In the end, due to the modest antitumor effect and limited effect on patient survival, bevacizumab is not recommended to be used in the first-line setting to treat metastatic TNBC.

### 6.3. PI3K/AKT/mTOR Targeted Therapy in TNBC

The phosphoinositide-3 kinase (PI3K) and AKT signaling pathways are potentially actionable targets in TNBC. Activating mutations in these signaling pathways, such as *PIK3CA* and *AKT1*, occur in about 25% of primary TNBC [225,226,227]. Additionally, PI3K inhibitors have shown some promising efficacy in stage II-III TNBC patients whose tumors have *PIK3CA* mutations [68,207,225]. Following on the efficacy of alpelisib to improve PFS for HR-positive breast cancer [228,229,230], alpelisib plus nab-paclitaxel is being assessed in anthracycline-refractory TNBC with *PIK3CA* or *PTEN* mutations in a phase II trial [225]. The addition of everolimus, an mTOR inhibitor, was found to be synergistic with cisplatin and paclitaxel in the treatment of stage II/III TNBC patients. However, significant side effects and adverse events were also observed in the everolimus arm, without any improvement in pCR or clinical response in this randomized phase II neoadjuvant study [68]. Since the mesenchymal subtype of TNBC is often associated with aberrant PI3K/mTOR pathway activation, increased invasion, and poor outcomes, the addition of temsirolimus or everolimus has been tested in combination with liposomal doxorubicin and bevacizumab. The addition of the mTOR inhibitor to treat metaplastic TNBC resulted in a significant improvement in objective response rate (31% vs. 0%; *p* = 0.04) but not in clinical benefit rate (44% vs. 45%; *p* > 0.99) in these patients whose TNBC tumors showed increased PI3K pathway activation [231]. The AKT inhibitors, like ipatasertib and capivasertib, have shown promise in improving outcomes for patients with high-risk TNBC [207,232,233]. The LOTUS trial (NCT02162719) was a randomized, double-blind, phase II study on 124 treatment-naïve patients with inoperable, locally advanced, or metastatic TNBC. Patients enrolled in the study were randomly assigned (1:1) to be treated with paclitaxel plus either ipatasertib or a placebo. The study reported that mTNBC patients who were treated with ipatasertib had an improved median PFS compared to the placebo (6.2 months vs. 4.9 months, respectively, *p* = 0.037). In the subset of mTNBC patients with *PIK3CA*/*AK1*/*PTEN* mutations, patients treated with ipatasertib had a median PFS of 5.3 months compared to 3.7 months in patients treated with the placebo (*p* = 0.36) [234]. The PAKT trial (NCT02423603), a phase 2 randomized and double-blind study, tested the efficacy of capivasertib with paclitaxel compared to a placebo and paclitaxel in 140 patients with untreated mTNBC [235]. The addition of capivasertib improved median PFS slightly (5.9 months vs. 4.2 months) and OS (19.1 months vs. 12.6 months) compared to the paclitaxel arm alone. The benefits of capivasertib were more pronounced in the subset of TNBC patients with *PIK3CA*/*AK1*/*PTEN* mutations (n = 28), and these specific TNBC patients treated with capivasertib and paclitaxel had a median PFS of 9.3 months compared to a median PFS of 3.7 months for patients treated with a placebo and paclitaxel [235].

### 6.4. AR Targeted Therapy in TNBC

AR is a nuclear steroid hormone receptor that is expressed at a variety of levels in 10–43% of TNBC [68,227,236]. The relationship between AR expression and prognosis for TNBC patients remains unclear and controversial. For some patient populations in the United States and Nigeria, AR expression is associated with a favorable outcome. However, for patients in other countries, such as Norway and India, AR expression is associated with a poor outcome [237]. Several phase 2 clinical trials have been conducted to test the clinical efficacy of multiple FDA-approved AR inhibitors for AR-positive prostate cancer as a possible treatment for AR-positive TNBC. In the first phase 2 study of metastatic AR-positive TNBC breast cancer patients, bicalutamide, an AR antagonist, showed a six-month clinical benefit rate of 19% [95% CI, 7–39%] and a median PFS of 12 weeks (95% CI, 11–22 weeks) [238]. In another phase 2 single-arm trial (UCBG 12-1), a different AR inhibitor, abiraterone acetate plus prednisone, was used to treat a cohort of 146 AR-positive TNBC patients with inoperable locally advanced or metastatic diseases whose tumors had ≥ 10% AR expression. This study showed a six-month clinical benefit rate of 20% [95% CI, 7.7–38.6%] and a median PFS of 2.8 months (95% CI, 1.7–5.4%) for abiraterone, which was comparable to bicalutamide [239]. In a third phase 2 single-arm and two-stage trial (MDV3100-11), another potent AR inhibitor, enzalutamide, was used to treat a cohort of 118 AR-positive TNBC patients: 78 of these TNBC tumors had ≥ 10% AR expression (AR-High) and 40 of these TNBC tumors had ≥ 0% AR expression (AR-Low). The AR-High TNBC patients who received enzalutamide had a 16-week clinical benefit rate of 33% (95% CI, 23–45), a median PFS of 3.3 months (95% CI, 1.9–4.1), and a median OS of 16.5 months (95% CI, 12.7–20.0). Patients with AR-Low TNBC tumors who received enzalutamide had a 16-week clinical benefit rate of 25% (95% CI, 17–33), a median PFS of 2.9 months (95% CI, 1.9–3.7), and a median OS of 12.7 months (95% CI, 8.5–16.5) [240,241]. However, given the unclear relationship between AR expression and prognosis for the AR-positive TNBC cohort, it is uncertain whether the clinical benefits from AR inhibitors like bicalutamide, abiraterone, and enzalutamide should be attributed to the anti-AR treatments or to the overall favorable outcomes for the AR-positive TNBC subset. Therefore, additional studies are required to demonstrate whether AR expression is a useful prognostic biomarker and an actionable drug target in mTNBC prior to the incorporation of AR inhibitors in the treatment of AR-positive mTNBC [39,242]. One such study is the START trial (NCT03383679), an ongoing randomized phase 2 study testing the efficacy of darolutamide, a new AR antagonist, compared to capecitabine for AR-positive, locally recurrent (unresectable), or metastatic TNBC (https://clinicaltrials.gov/ct2/show/study/NCT03383679).

### 6.5. ERβ Targeted Therapy in TNBC

Estrogen receptor beta (ERβ) is highly expressed in normal mammary tissue [243,244,245]. ERβ expression level is gradually decreased or completely lost during mammary tumorigenesis in a variety of highly aggressive and malignant breast cancers [244,246,247,248]. As a known tumor suppressor, persistent ERβ expression is associated with a less aggressive and non-invasive phenotype, and prolonged patient survival [249]. ERβ expression is retained in 30% of TNBCs, whereas its expression is lost in 70% of TNBC [250,251]. For ERβ-positive TNBC cells, adding estrogen (E2) or other ERβ-selective agonists to activate ERβ receptor can elicit potent anticancer effects by inducing cystatin gene expression, decreasing cell proliferation, inhibiting canonical TGFβ pathway activation, blocking epithelial-to-mesenchymal transition, and preventing malignant cell invasion and metastatic spread [252,253,254,255,256,257]. These results suggest that ERβ-augmentation therapies can elicit tangible clinical benefits for a subset of ERβ-positive TNBC tumors with a good prognosis [258,259]. One limitation is that anti-ERβ therapy will not benefit ERβ-negative TNBC patients with an aggressive phenotype and poor prognosis.

## 7. K-RAS/SIAH is a Major Tumor-Driving Signaling Pathway in TNBC

### 7.1. SIAH’s Gatekeeper Role is Indispensable for Proper K-RAS/EGFR Signal Transduction

**Figure 1:**
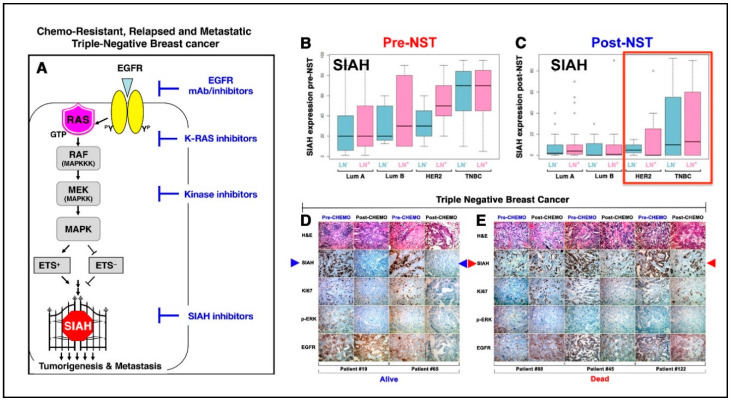
SIAH^ON/OFF^ binary expression in residual tumors post-neoadjuvant systemic therapy (NST) can be used to risk-stratify pIR patients and predict patient survival in high-risk TNBC at 5 years. (**A**) Schematic illustration of the K-RAS–SIAH–EGFR pathway activation in TNBC. SIAH is the most downstream “gatekeeper” signaling module in the canonical K-RAS/EGFR signal transduction pathway in TNBC. (**B**,**C**) Loss of SIAH expression is correlated with K-RAS pathway inactivation and tumor regression, whereas persistent SIAH expression is correlated with K-RAS activation and tumor relapse in breast cancer of mixed molecular subtypes. The box-and-whisker plots were used to graphically illustrate the population distribution of median SIAH expression levels in both node-positive (as marked by purple color bar graphs) and node-negative (as marked by teal color bar graphs) in breast cancer of the four molecular subtypes: Luminal A (LumA), Luminal B (LumB), HER2, and TNBC. (**B**) The median SIAH expression levels in the untreated node-negative and node-positive primary tumors of the 4 molecular subtypes pre-NST are shown: Luminal A (LN-negative LumA at 20% and LN-positive LumA at 20%), Luminal B (LN-negative LumB at 20% and LN-positive LumB at 30%), HER2-positive breast cancer (LN-negative HER2-positive breast cancer at 30% and LN-negative HER2-positive breast cancer at 50%), and TNBC (LN-negative TNBC at 70% and LN-positive TNBC at 70%). The data showed that TNBC has the highest proliferative index in a peerwise comparison. (**C**) The median SIAH expression levels in the treated node-negative and node-positive residual tumors of the 4 molecular subtypes post-NST are shown: Luminal A (LN-negative LumA at 2% and LN-positive LumA at 3%), Luminal B (LN-negative LumB at 0.5% and LN-positive LumB at 1%), HER2-positive breast cancer (LN-negative HER2-positive breast cancer at 3% and LN-negative HER2-positive breast cancer at 0.5%), and TNBC (LN-negative TNBC at 8% and LN-positive TNBC at 15%). The data showed that TNBC is a high-risk cohort with intrinsic chemo-resistance, independent of the LN status, in a group comparison. The error bars or whiskers in the histogram and bar charts represent the 95% CI, and in the box plots, they represent the upper (top) and lower quartiles (bottom) data distribution—with points beyond 95% CI representing the outliers. Importantly, SIAH^ON^ expression can be used to accurately identify the individual pIR outliers with high SIAH expression and poor survival in breast cancer. (**D**,**E**) Representative IHC images of SIAH, EGFR, phospho-ERK, and Ki67 staining in TNBC pIR residual tumors are shown. (**D**) The pIR patients with no or low SIAH expression in residual tumors post-NACT stayed in remission. SIAH^OFF^ marked chemo-sensitive TNBC tumor cells that have stopped growing post-NACT, predicting increased patient survival (**Alive**). (**E**) The partial responders with high SIAH expression in residual tumors (despite 90% tumor shrinkage) post-NACT developed tumor relapse and succumbed to their metastatic diseases. SIAH^ON^ identified chemo-resistant TNBC tumor cells that are still growing post-NACT, thus predicting poor survival (**Dead**). **Conclusion**: For TNBC pIR patients with 70–90% tumor reduction post-NACT, it is evident that persistent high SIAH expression in residual tumors will predict early tumor relapse, poor prognosis, and reduced survival, whereas no or low SIAH expression in residual tumors will predict tumor remission, good prognosis, and increased survival in both the node-negative and node-positive TNBC post-NACT.

Normal K-RAS/SIAH/EGFR signaling pathway activation is indispensable for proper cellular communication, cell proliferation, and tissue homeostasis in multicellular organisms. However, abnormal K-RAS/SIAH/EGFR pathway activation is highly prevalent in chemo-resistant, recurrent, and metastatic TNBC [141,142,260,261,262,263,264,265]. Seven in absentia homologue (SIAH) RING-domain E3 ligase is the most downstream signaling gatekeeper and the most evolutionarily conserved signaling molecule in the EGFR/HER2/K-RAS signaling pathway (Figure 1A) [19,141,142,266,267]. Based on its extraordinary evolutionary conservation and high significance as the most downstream signaling “gatekeeper” required for proper K-RAS/EGFR signal transduction, SIAH^ON/OFF^ is a binary code whose expression is a reliable readout of EGFR/RAS/RAF/MEK/MARK pathway activation/inactivation in human cancer. Supported by strong evidence in developmental, evolutionary and cancer biology, we hypothesize that K-RAS/SIAH pathway activation is a major tumor driver, and SIAH represents a strategically well-positioned tumor vulnerability and a new therapeutic target against chemo-resistant, relapsed, and metastatic TNBC in the future (Figure 1A).

### 7.2. K-RAS/SIAH/EGFR Pathway is Commonly Activated in TNBC, and SIAH is a Therapy-Responsive and Prognostic Biomarker in TNBC

Genomic landscape studies have indicated that activation of the tumor-driving K-RAS/EGFR pathway is highly prevalent in high-grade, locally advanced, relapsed, and chemo-refractory TNBC [208,268,269,270,271,272,273,274]. Furthermore, we and others have shown that K-RAS/SIAH pathway activation is associated with progression of DCIS to invasive ductal cancer, and reduced survival of luminal-type breast cancer [262,275]. Hence, studying activation/inactivation of the tumor-driving K-RAS/SIAH/EGFR pathway represents an opportunity to define therapy-responsive and prognostic K-RAS/SIAH-centered biomarkers in TNBC. This SIAH-centered anti-TNBC strategy may provide a solid foundation on which to stratify TNBC partial responders, identify chemo-refractory tumors, predict survival, and decide whether to add adjuvant therapies to control chemo-resistant residual TNBC post-NACT.

SIAH^ON^ expression indicates persistent EGFR/RAS/RAF/MEK/MAPK pathway activation and cancer cell proliferation and predicts for tumor progression, whereas SIAH^OFF^ expression indicates EGFR/RAS/RAF/MEK/MARK pathway inactivation, diminished cell proliferation, and tumor regression [142,276]. As a binary code (SIAH^ON/OFF^) to predict tumor progression/regression post-NACT, SIAH is a useful prognostic biomarker in TNBC [142,266,267] (Figure 1). We found that persistent high expression of SIAH in residual tumors reflects activation of the “tumor-driving” K-RAS/SIAH/EGFR pathway that fuels tumor growth and metastatic spread of disseminated and residual chemo-resistant tumor clones remaining after NACT (Figure 1B–E) [276]. Currently, there are no reliable prognostic molecular biomarkers that can be used to risk-stratify pIR patients, identify chemo-resistant tumor clones, quantify tumor response, forecast early tumor relapse, and predict patient survival after surgical tumor resection post-NACT in TNBC. We hypothesize that SIAH is well positioned to serve as a new biomarker whose ON/OFF expression can be used to predict TNBC recurrence/remission post-NACT. By comparing the percentage reduction (%) of SIAH expression in primary mammary tumors pre- and post-NACT, SIAH could potentially be used to quantify the efficacy of chemotherapy, identify chemo-resistant residual tumors, and forecast early tumor relapse post first-line chemotherapy. Conversely, the SIAH^ON/OFF^ binary code classification in the residual tumor at a single tumor cell resolution could potentially augment prognosis and permit accurate risk stratification of low-risk pIR patients who are likely to stay in remission and thus may not need additional adjuvant therapy from high-risk pIR patients who are destined to relapse post-NACT and could benefit from additional adjuvant therapies (Figure 1).

### 7.3. SIAH as an Actionable Target Against EGFR-Driven TNBC.

The EGFR pathway activation remains a major drug target in TNBC. EGFR is upregulated and overexpressed in approximately 50% of TNBC patients [213,260,276]. Although the anti-EGFR monoclonal antibody, cetuximab, was ineffective in shutting down EGFR activation in TNBC, this does not mean that inhibiting the EGFR pathway activation in some other ways might not impede EGFR-driven TNBC tumorigenesis and metastasis. The lack of efficacy of anti-EGFR therapy in TNBC may be attributed to the compensatory co-activation and extensive network crosstalk of multiple effector pathways downstream of EGFR, that drive aggressive tumorigenesis and metastatic dissemination of TNBC. EGFR activation signals through the K-RAS/SIAH signaling pathway [263,277,278,279,280,281,282]. Based on the aggressive TNBC tumor biology and high proliferation indices of most TNBC, we hypothesize that EGFR-driven TNBC tumors are uniquely suited for a SIAH-centered biomarker discovery program as well as development of anti-SIAH-based targeted therapy by targeting this conserved and essential signaling bottle neck, SIAH, to shut down this highly adaptive EGFR/RAS/RAF/MEK/MARK signaling network that drives chemo-resistant, relapsed, and metastatic TNBC in the clinic (Figure 1).

In the future, we hope to develop a companion computer algorithm by incorporating the SIAH^ON/OFF^ binary code to augment RCB risk stratification, and triage and differentiate high-risk pIR TNBC patients who are likely to develop early tumor relapse from lower-risk pIR patients who are likely to stay in remission post-NACT. Ultimately, developing a new SIAH pathway-centered prognostic biomarker panel and a novel anti-SIAH-based targeted therapy for the highest-risk TNBC patients could be very important. Additionally, future research should focus on SIAH and SIAH-interacting proteins as additional actionable targets in TNBC by conducting global signaling pathway analysis through reverse phase protein microarrays (RPPA) and phosphoproteomic profiling of cancer kinomic signaling pathways in multidrug-resistant, relapsed, and late-stage TNBC.

## 8. Concluding Remarks

Chemo-refractory and metastatic TNBC is a major health challenge, resulting in high relapse rates and poor survival [16]. NACT is standard treatment for women with high-risk TNBC. A completed course of NACT results in two possible outcomes: pCR or pIR with residual disease. In general, pCR patients do well, whereas pIR patients exhibit dramatically different clinical outcomes which can be predicted, albeit based on statistical modeling, by use of the RCB classification. However, given that NACT regimens may take up to four–six months to complete, new biomarkers are needed to identify TNBC patients that are unlikely to respond to standard approaches, given the dismal prognosis for chemo-resistant TNBC. Thus, developing new, interactive, therapy-responsive, and prognostic biomarkers to further risk-stratify pIR patients with residual disease in real time during or after NACT are needed in order to identify patients at the highest risk for tumor recurrence, and to develop actionable therapeutic targets to prevent emergence of metastatic disease and eradicate multidrug-resistant, relapsed, and inoperable mTNBC.

The treatment disparity in TNBC stems from its genetic diversity, tumor/TME heterogeneity, and the lack of curative therapies in resistant, recurrent, and metastatic settings. The lack of expression of ER, PR, and HER2-neu limits standard TNBC treatment to multiple regimens of cytotoxic chemotherapies. Alongside currently FDA-approved new approaches targeting the host immune tumor surveillance system (anti-PD-1/anti-PD-L1), the already compromised DNA repair machinery with *BRCA*1/2 mutations (PARP inhibitors), and topoisomerase I inhibitors (sacituzuamb), a logical next opportunity is to target SIAH in the K-RAS/EGFR pathway in malignant TNBC. SIAH is essential for proper K-RAS/EGFR signaling pathway activation. SIAH^ON^ expression indicates EGFR/RAS/RAF/MEK/MAPK pathway activation and tumor progression, whereas a lack of SIAH expression, SIAH^OFF^, indicates EGFR/RAS/RAF/MEK/MAPK pathway inactivation and tumor regression post-NACT. As such, SIAH is well positioned to become a new tumor-specific, therapy-responsive, and prognostic biomarker, and a major tumor vulnerability, and a new therapeutic target in TNBC (Figure 1). Targeted SIAH therapies in conjunction with surgery, chemo-, radiation, targeted, and immune checkpoint blockade therapy may improve the outcomes of TNBC patients in the future. Further detailed studies are required to delineate the biological function, substrate selection, target degradation, molecular regulation, signaling rewiring, and crosstalk of the SIAH/K-RAS/EGFR pathway in the context of a dynamic and heterogeneous TNBC signaling network in vitro and in vivo.

Focusing on the K-RAS/SIAH pathway should bring much-needed attention to this important and evolutionarily conserved tumor-driving pathway that fuels chemo-resistant TNBC. Although the role of oncogenic K-RAS pathway activation has been well established in several of the deadliest cancer types, its mechanism of activation in chemo-resistant, relapsed, and metastatic TNBC remains elusive. This lack of mechanistic understanding along with the low mutation rate of K-RAS in breast cancer may contribute to it being understudied in this high-risk population. The K-RAS/SIAH pathway is nonetheless an important area of investigation, with the potential to reveal biomarkers that would permit better assessment for real-time clinical decision-making during and after NACT of TNBC. SIAH has shown a good clinical promise to stratify TNBC pIR patients and augment RCB classification post-NACT (Figure 1). The discovery and validation of therapy-responsive and prognostic K-RAS/SIAH/EGFR pathway biomarkers is an important development in TNBC. Ultimately, the hope is to translate SIAH into clinical practice to detect ineffective chemotherapy, identify chemo-resistant tumor clones, forecast early tumor relapse, and predict outcome and survival as early as possible. New targeted therapy that blocks SIAH function, possibly combined with chemo-, radiation, and targeted therapy and/or immune checkpoint blockade treatment, may improve the outcomes of a subset of TNBC patients whose invasive residual tumors retain a high-proliferation index post-NACT. We strongly encourage the development of new anti-SIAH-centered anti-EGFR/RAS/RAF/MEK/MARK targeted therapy to treat chemo-resistant, locally advanced, and metastatic TNBC in the hopes of saving more lives in the future.

## Abbreviations

ACT	Adriamycin: Cytoxan, and Taxotere
AR	Androgen receptor
CI	confidence interval
DFS	disease-free survival
DRFS	distant recurrence-free survival
EGFR	epidermal growth factor receptor
ER	estrogen receptor
HER2	human epidermal growth factor receptor 2
H&E	hematoxylin and eosin staining
HR	Hazard Ratio
IDFS	invasive disease-free survival
IHC	immunohistochemistry
LN	lymph node
MBC	metastatic breast cancer
mTNBC	metastatic TNBC
mTOR	the mammalian target of rapamycin
NACT	neoadjuvant chemotherapy
OS	overall survival
PARP	poly-ADP-ribose polymerase
pCR	pathological complete response
PD-1	programmed cell death receptor-1
PD-L1	programmed death ligand-1
PFS	progression-free survival
PI3K	phosphoinositide-3 kinase
pIR	pathological incomplete response
PKB	protein kinase B (AKT)
PR	progesterone receptor
RCB	Residual Cancer Burden
SEER	Surveillance, Epidemiology and End Results Program
SIAH	human homologues of Drosophila Seven-In-Absentia
SOC	standard of care
TIL	tumor infiltrating lymphocytes
TME	tumor microenvironment
TNBC	triple-negative breast cancer
TNM	tumor size, lymph node status, metastasis
Trop-2	Trophoblast cell-surface antigen
